# Bridging Innovation Research with Clinical Application: Clinical Trials at the Institute of Hematology & Blood Diseases Hospital, Chinese Academy of Medical Sciences

**DOI:** 10.1055/s-0044-1788572

**Published:** 2024-07-12

**Authors:** Xiaochen Wang, Lijun Liu

**Affiliations:** 1 State Key Laboratory of Experimental Hematology, National Clinical Research Centre for Blood Diseases, Haihe Laboratory of Cell Ecosystem, Institute of Hematology & Blood Diseases Hospital, Chinese Academy of Medical Sciences & Peking Union Medical College, Tianjin, China; 2 Tianjin Institutes of Health Science, Tianjin, China

Clinical trials provide fundamental data in developing clinical application of investigational drugs, medical devices, and medical technology products. As the largest specialized hospital in hematology, the Institute of Hematology & Blood Diseases Hospital, Chinese Academy of Medical Sciences (IHCAMS) founded the first clinical trial center in hematology in China in 1983. In addition to supporting the approval of the first chimeric antigen receptor T-cell therapy (CAR-T) product (CNCT19) for acute myeloid leukemia (AML) by the National Medical Products Administration (NMPA) of China in 2023, the first registered clinical trial of adeno-associated virus (AAV) gene therapy for hemophilia, the first cell therapy for beta-thalassemia, the first mesenchymal stem cell (MSC) therapy for graft versus host disease (GVHD) and immune thrombocytopenia (ITP), and the first nonviral CAR-T therapy for lymphoma in China were all led by the IHCAMS.

Since 2013, 895 clinical trials for new drugs in hematological diseases have been registered at the Center for Drug Evaluation (CDE), NMPA. Among them, the IHCAMS is involved in 460 projects, including 145 projects in the Lymphoma and Myeloma Center, 97 projects in the GCP (Good Clinical Practice) center, 91 projects in the Hemophilia Center, 47 projects in the Leukemia and Immunotherapy Center, 28 projects in the MDS Center, 23 projects in the Transplantation Center, 19 projects in the Anemia Center, 8 projects in the Pediatrics Center, and 2 projects in the Regenerative Medicine Center. IHCAMS is the leading organization for clinical trials of new drugs in haematological diseases with 232 clinical trials; this is at least twice the number at other clinical centers.

Recently, results from several clinical centers from the IHCAMS were reported, covering multiple hematological diseases.

AML is the most common type of acute leukemia in adults, with 2 to 10% of patients in China associated with mutations in the gene encoding isocitrate dehydrogenase 1 ( *IDH1* ). Ivosidenib (an oral small molecule inhibitor of mutant IDH1) monotherapy was approved by the U.S. Food and Drug Administration in 2019 for the treatment of relapsed or refractory (R/R) adult AML patients with IDH1 mutation. To evaluate the pharmaceutical characteristics, safety, and clinical efficacy of ivosidenib, a bridging study, CS3010-101 (NCT04176393), was conducted by Dr. Jiangxiang Wang's group at the Leukemia Center of the IHCAMS. 1 The study showed results consistent with those of the pivotal study, suggesting the potential of ivosidenib as a single-agent therapy for Chinese AML patients.

To target the R/R multiple myeloma (MM) patients in China, subcutaneous daratumumab, a human immunoglobulin G kappa (IgGκ) monoclonal antibody targeting CD38, was evaluated at the Lymphoma and Myeloma Center through a phase 1, multicenter study (MMY1010; ClinicalTrials.gov Identifier: NCT04121260). 2 Led by Dr. Lugui Qiu's group at the IHCAMS, eligible patients from five hospitals in China participated in this clinical trial. The results show that the subcutaneous formulation of daratumumab produces similar results in Chinese patients as those observed in the United States, Europe, and Japan, supporting its clinical potential in the Chinese population.

Other than clinical trials for traditional drugs, a number of Investigational New Drug (IND) applications were approved for stem cell and gene therapies. The effectiveness of MSCs in treating hematologic malignancies have been reported, with more and more MSC products approved worldwide. Recently, a phase I trial of human umbilical cord–derived mesenchymal stem cells (UC-MSCs) was reported for the first time in the treatment of refractory ITP by Lei Zhang's group of Hemophilia Center (ClinicalTrials.gov ID: NCT04014166). 3 More evidence is needed to assess the efficacy and safety of this innovative therapy, in which the mechanism studies need to be further explored. Nevertheless, this therapy appears to be an effective alternative treatment for this immune disease.

## References

[1] Sun M Yin Q Liang Y Ivosidenib in Chinese patients with relapsed or refractory isocitrate dehydrogenase 1 mutated acute myeloid leukemia: a registry study Blood Sci 2024 6 03 e00196 38911469 10.1097/BS9.0000000000000196 PMC11191922

[2] An G Ge Z Jing H Subcutaneous daratumumab in Chinese patients with relapsed or refractory multiple myeloma: an open-label, multicenter, phase 1 study (MMY1010) Blood Sci 2024 6 03 e00193 38832105 10.1097/BS9.0000000000000193 PMC11146469

[3] Chen Y Xu Y Li H A novel anti-CD38 monoclonal antibody for treating immune thrombocytopenia N Engl J Med 2024 390 23 2178 2190 38899695 10.1056/NEJMoa2400409

